# Human Papillomavirus Vaccine Impact and Effectiveness in Six High-Risk Populations: A Systematic Literature Review

**DOI:** 10.3390/vaccines10091543

**Published:** 2022-09-16

**Authors:** Elizabeth Goodman, Miriam Reuschenbach, Allysen Kaminski, Sarah Ronnebaum

**Affiliations:** 1 Center for Observational and Real-World Evidence, Merck & Co., Inc., Rahway, NJ 07065, USA; 2 Global Medical and Scientific Affairs, MSD Sharp and Dohme GmbH, 81673 Munich, Germany; 3 OPEN Health, Bethesda, MD 20814, USA

**Keywords:** HPV, HPV vaccination, vaccine effectiveness, vaccine impact, real-world evidence, systematic literature review

## Abstract

Specific adult populations known to be at high risk for human papillomavirus (HPV)-related disease, such as men who have sex with men, are inconsistently included in national immunization programs. No compilation of the evidence on the real-world impact and effectiveness of HPV vaccines across these populations exists. This systematic literature review identifies and synthesizes the evidence of the real-world impact and effectiveness of the quadrivalent and nonavalent HPV vaccines in high-risk populations: women with prior/current HPV-related anogenital disease, men who have sex with men, immunocompromised/immunosuppressed individuals, female sex workers, transgender and non-binary individuals, and patients with recurrent respiratory papillomatosis (RRP). The outcomes included anogenital precancers/cancers, head and neck cancers, genital warts, and RRP recurrence. From the 2216 records identified, 30 studies (25 effectiveness and 5 impact studies) were included in this systematic literature review. The results, quantity, and quality of these studies were highly variable. The evidence for effectiveness was of high quality only in women with prior/current cervical disease and in individuals with RRP, the most frequently studied populations. No studies of transgender/non-binary individuals or female sex workers were identified. The real-world evidence supports HPV vaccination among women with prior cervical disease and individuals with RRP. Significant real-world data gaps remain in these high-risk populations.

## 1. Introduction

Human papillomavirus (HPV) is the most common sexually transmitted infection globally and causes a number of serious diseases, such as cervical and anal cancers, genital warts, and recurrent respiratory papillomatosis (RRP). Globally, HPV is thought to contribute to nearly 600,000 new cancer cases and over 300,000 premature deaths annually [1]. Vaccination to protect against HPV-related disease has been available since 2006 when Gardasil^®^ (human papillomavirus quadrivalent vaccine [4vHPV], recombinant; Merck & Co., Inc., Rahway, NJ, USA) which targets HPV types 6, 11, 16, and 18, was approved in the United States and Europe [2]. Gardasil^®^9 (human papillomavirus 9-valent vaccine [9vHPV], recombinant; Merck & Co., Inc., Rahway, NJ, USA) targets five additional HPV genotypes (31, 33, 45, 52, and 58) and was approved in 2014 in the United States and a year later in Europe [2]. In addition to 4vHPV and 9vHPV, there is a bivalent (2vHPV) vaccine which targets HPV types 16 and 18 (Cervarix^®^; GSK, Rixensart, Belgium) which was licensed in 2007 in Europe and 2009 in the United States [2]. The results of a global study of over 18,000 HPV-related cancer specimens suggest that the 9vHPV vaccine can prevent 90% of cervical, 79% of anal, 61% of vaginal, 25% of penile, 23% of vulvar, 21% of oropharynx, 4% of oral cavity, and 3% of larynx cancer cases [3]. As of June 2020, 55% of WHO member states had fully or partially incorporated HPV vaccines into their national immunization programs [4].

There is growing evidence of the real-world impact and effectiveness of 4vHPV and 9vHPV in reducing HPV-related disease. More than 125 studies from 22 countries on the real-world impact and effectiveness of vaccination have been published. Most real-world impact and effectiveness studies focus on the impact and effectiveness of vaccination in the general population [5,6,7,8,9]. However, several also focus on populations known to be at high risk for HPV-related disease, such as women who have received excisional therapy for cervical intraepithelial neoplasia or men who have sex with men (MSM). Studies focusing on specific high-risk populations have been summarized in previous systematic literature reviews (SLRs) [10,11,12,13,14,15,16,17,18]. To date, there has been no real-world evidence synthesis across multiple high-risk populations, particularly those high-risk sub-groups that have been included or are being considered for inclusion in some national immunization programs. The objective of the present SLR was to address this evidence gap by systematically identifying and synthesizing real-world evidence of the effectiveness and impact of 4vHPV and 9vHPV across a broad range of HPV-related diseases in specific high-risk populations that generally do not have access to HPV vaccination through national immunization programs.

## 2. Methods

This SLR included studies of six populations at high risk for HPV-related disease: (1) women with pre-existing or current HPV-related anogenital diseases; (2) MSM; (3) immunocompromised or immunosuppressed individuals; (4) female sex workers; (5) transgender and non-binary individuals; and (6) patients with RRP. Studies of the following HPV-related disease outcomes were included: anogenital precancers/cancers (cervical, vulvar, vaginal, penile, and anal precancers/cancers); head and neck cancers; and genital warts. HPV infection was not included as an outcome. For RRP patients, the intersurgical interval, number of surgeries per month, and recurrent disease outcomes were used to assess vaccine effectiveness at reducing the disease burden in this population. The SLR was conducted according to Preferred Reporting Items for Systematic Reviews and Meta-Analyses (PRISMA) guidelines and guidelines from the Cochrane Group. This study was registered with PROSPERO (registration number CRD42021245948). Ethics committee approval was not required for this study.

### 2.1. Study Inclusion Criteria and Search Strategy

The selection criteria for this SLR were based on the PICOTS (population, interventions, comparisons, outcomes, timing, and setting) framework (Table 1) [19]. Using prespecified search terms (Supplementary Materials Table S1), MEDLINE and Embase databases were queried on 2 February 2021 using the ProQuest platform. The search included articles published at any time and conference abstracts published in 2018 or later. Exclusion criteria included non-English publications, duplicate publications, conference abstracts published prior to 2018, clinical trial study designs, decision analytic and cost-effectiveness modeling studies, use of 2vHPV in the majority of vaccinated subjects in the study population, and vaccine impact studies of juvenile onset RRP, as such impact is a consequence of the childhood vaccination of girls, the primary vaccination cohort, and not of the vaccination of a high-risk population.

### 2.2. Data Extraction and Data Analysis

The data extracted for each study included study characteristics (citation information, design, location, inclusion/exclusion criteria, data source and collection dates), subject characteristics (age, sex, race/ethnicity, body mass index, HPV DNA/disease information [before and/or after treatment, including DNA presence, cytology, and histology]) vaccination information (distribution setting, type, doses, timing relative to treatment) and disease outcomes by vaccination status for vaccine effectiveness studies and by time period for vaccine impact studies. The risk of bias in each included study was assessed using the Newcastle-Ottawa scale [20,21]. The quality of the totality of evidence for each outcome and high-risk population was assessed and reported using the 4-point scale provided by the Grading of Recommendations, Assessment, Development and Evaluation (GRADE) working group [22]. Descriptive statistics were reported at the aggregate level. For RRP studies that presented patient-level but not aggregate data, the mean, median, and standard deviation (SD) were calculated using patient-level data, but no statistical tests were performed. Due to heterogeneity in reported outcomes, cervical precancers of grade cervical intraepithelial neoplasia (CIN) 2 and higher (CIN2, CIN3, carcinoma in situ [CIS], and high-grade squamous intraepithelial lesion [HSIL]) are labeled ‘CIN2+’ throughout this report. Herein, vaccine effectiveness estimates are reported for statistically significant (*p* < 0.05) results.

## 3. Results

**Figure 1:**
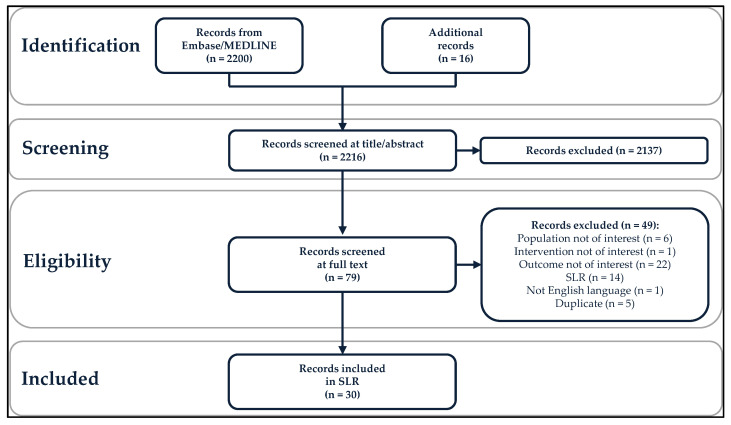
Preferred Reporting Items for Systematic Reviews and Meta-Analyses (PRISMA) diagram. SLR, systematic literature review.

A total of 2200 records were identified from the literature search in Embase/MEDLINE, and 16 additional records were included for review (Figure 1). The PICOTS criteria were applied in a two-step process: 2137 records were excluded in the title/abstract review phase, and 49 records were excluded following review of the full text, leaving 30 included records [17,23,24,25,26,27,28,29,30,31,32,33,34,35,36,37,38,39,40,41,42,43,44,45,46,47,48,49,50,51]. Included studies were conducted in 15 countries: Australia, Brazil, Canada, Czech Republic, Denmark, Germany, Italy, Japan, the Netherlands, Romania, Slovenia, South Korea, Spain, the United Kingdom, and the United States. Vaccine effectiveness studies were more common than vaccine impact studies (25 versus 5 studies). Individuals with RRP were the most studied population (12 vaccine effectiveness studies), followed by women with pre-existing or current HPV-related anogenital disease (eight vaccine effectiveness studies), MSM (three vaccine effectiveness and five vaccine impact studies), and immunosuppressed or immunocompromised individuals (two vaccine effectiveness studies). No studies related exclusively to transgender and non-binary populations or sex workers were found, although a small number of transgender women were included in a study of MSM [48].

### 3.1. Vaccine Use in Included Studies

Vaccine use was reported in all studies. 4vHPV was the sole vaccine provided in 11 of the 12 RRP studies [17,26,32,33,34,37,38,39,41,49,51], all studies of immunocompromised/immunosuppressed individuals [40,45], all vaccine impact studies of anogenital warts in MSM [23,25,27,29,36], and two of the three vaccine effectiveness studies in MSM [46,47]. One of the vaccine effectiveness studies in MSM used 4vHPV in conjunction with 9vHPV [48], as did one of the RRP effectiveness studies [50]. Among the three included studies that used both 4vHPV and 2vHPV, all were among women with prior anogenital disease [24,42,43]. 2vHPV usage ranged from 2% [43] to 47% [42]. Del Pino 2020 included all three vaccines, with 64.1% receiving 9vHPV, 19.6% receiving 2vHPV, 4.6% receiving 4vHPV, and the remainder (11.7%) unknown [28]. Among 21 studies that reported the number of received vaccine doses [24,28,30,31,32,33,34,35,37,38,39,40,42,45,46,47,48,49,50,51], 5% to 100% of the patients were fully vaccinated, with 15 studies (71.4%) reporting that 100% of the patient sample received three doses.

### 3.2. Effectiveness and Impact in High-Risk Groups

The results specific to each of the four risk groups for which studies were identified are presented below from most studied (RRP) to least studied (immunocompromised/immunosuppressed). Because no studies were identified for transgender/non-binary individuals or female sex workers, no results are presented for these populations.

### 3.3. Patients with RRP

**Figure 2:**
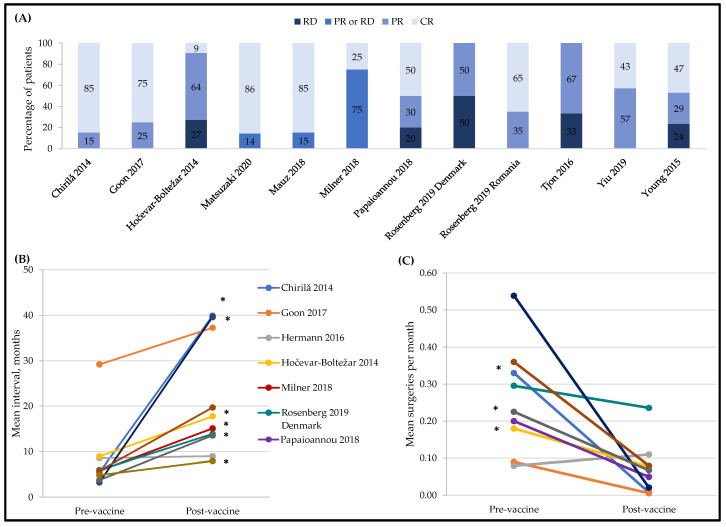
4vHPV vaccine effectiveness in patients with recurrent respiratory papillomatosis. Panel (**A**) shows the tumor response within an individual patient defined as: CR, complete response; PR, partial response; and RD, recurrent disease. Three studies defined PR as a >50% increase in the interval between surgical procedures [32,49,50]; one study as a >50% increase in the interval between surgical procedures or persistent papillomas that were not growing and did not require surgical interventions for >12 months [34]; one study as any increase in the time to recurrence [41]; and one study as >12 months with no appreciable growth of papillomas [51]. Three studies defined the ‘PR or RD’ category as any recurrence [37,38,39]. No definitions were provided in the remaining two studies [17,26]. These are descriptive, within person responses, so vaccine effectiveness is not calculated. Panel (**B**) shows the increase in the mean intersurgical interval. The symbol * indicates statistical significance at *p* < 0.05. Panel (**C**) shows the decrease in the mean number of surgeries per month. The symbol * indicates statistical significance at *p* < 0.05. For all panels, data from the Rosenberg 2019 Danish cohort were collected by the study authors while data from the Romanian cohort were new, never reported data from a prior published study [52].

Twelve studies of 13 small cohorts (defined as <50 participants) assessed the effectiveness of HPV vaccination at preventing recurrence and/or reducing the number of required treatments among people with RRP (Figure 2) [17,26,32,33,34,37,38,39,41,49,50,51]. All studies compared outcomes in the same patients before and after vaccination, and two studies also compared a vaccinated with an unvaccinated patient cohort [38,39]. Mauz 2018 compared 13 vaccinated to 11 unvaccinated RRP patients; 85.6% of the vaccinated achieved complete remission versus none of the unvaccinated, but no statistical significance testing was reported [38]. Milner 2018 compared 12 vaccinated to 16 unvaccinated RRP patients but did not demonstrate vaccine effectiveness. Complete remission was 25% in the vaccinated group compared to 15.8% in the unvaccinated group (*p* = NS), and the intersurgical interval was not significantly different between the groups (15.0 months vaccinated versus 16.6 months unvaccinated, *p* = NS) [39]. For all these RRP studies, non-significant results should be interpreted cautiously, as the small number of subjects studied lowered the power and increased the likelihood of a type 2 error.

Tumor response was reported in all RRP studies except Hermann 2016 [33] (Figure 2, Panel A). The studies differed in the timing of vaccine administration with respect to the most recent surgery and in the definition of partial tumor response. Papaioannou 2018 [41] used a more inclusive definition than other studies that described their criteria and two studies did not provide their definition of partial response [17,26]. Yiu 2019 described ‘remission’, but some participants in this category were listed as having post-vaccination surgical interventions, and vice versa [50]. Due to these discrepancies, two of the SLR authors determined the response category for members of this cohort with a partial response defined as a >50% increase in the intersurgical interval and a complete response defined as having no surgeries following the vaccination. Finally, pre-vaccination observation periods of up to 30 years that may have encompassed multiple changes in clinical practice were described in Tjon Pian Gi 2016 [49]. Mauz 2018 and Milner 2018 also compared the tumor response between vaccinated and unvaccinated patients, with the numbers suggestive of the protective effect of 4vHPV [38,39]. The intersurgical interval before and after HPV vaccination was reported for 10 cohorts [17,26,32,33,34,39,49,50,51], with the mean interval being numerically greater post-vaccination in all cohort except Hermann 2016 [33] (Figure 2, Panel B). These increases were statistically significant in six studies [17,26,34,39,50,51], while *p*-values were not reported for three studies [17,32,49].

The pre- and post-vaccination numbers of surgeries per month are shown in Figure 2, Panel C [17,26,32,33,34,41,49,50]. In eight of the nine cohorts, the number of surgeries per month was numerically lower following vaccination. This change was statistically significant in three cohorts [26,34,50]. For both the number of surgeries per month and intersurgical interval, the cohort with the shortest pre- and post-vaccination periods (12 months) was the only cohort for which the number of surgeries per month did not numerically decrease. In addition, Milner 2018 reported a decrease in the number of procedures that required pre- and post-vaccination during the 2–3 year observation period, but this decrease was not statistically significant (7.6 pre- versus 2.6 post-vaccination; *p* = 0.07) [39].

### 3.4. Women with Pre-Existing or Current Anogenital Disease

Eight studies assessed the effectiveness of HPV vaccination in women with various pre-existing HPV-related anogenital diseases; six among women with pre-existing cervical disease undergoing treatment [24,28,31,35,43,44], one on women with vulvar disease undergoing treatment [30], and one on women with genital warts [42]. All studies were based on reviews of records from clinics (single- or multi-center studies) except for Sand 2020 [44], which was a registry study.

**Figure 3:**
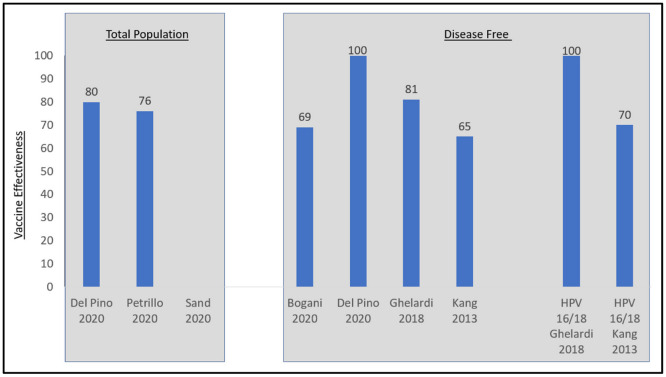
Vaccine effectiveness in women post excisional therapy for cervical disease. Disease-free is defined as those free of disease at 3 months in Kang, 2013 and free of disease at 6 months for Del Pino 2020, Ghelardi 2018, and Bogani 2020. Sources: Bogani 2020 [24], Del Pino 2020 [28], Ghelardi 2018 [31], Kang 2013 [35], Petrillo 2020 [43], and Sand 2020 [44].

Although the definitions and descriptions of persistent and recurrent disease differed across the six studies of women undergoing excisional treatment for cervical disease, five demonstrated vaccine effectiveness (Table 2, Figure 3). Four studies demonstrated effectiveness while adjusting for residual disease [24,28,31,35]. Petrillo 2020 did not adjust for residual disease but did show vaccine effectiveness [43]. Sand 2020 did not demonstrate vaccine effectiveness, but this study did not adjust for positive margins, a key factor in separating disease persistence from recurrence [44]. Three of these six studies stratified outcomes by HPV 16/18 type lesions versus non-vaccine covered HPV type lesions [28,31,35]. Kang 2013 found that among nine out of the 360 patients in the vaccinated group with recurrent CIN2+, five were recurrent HPV 16/18 and four of whom had cone margin involvement [35]. In subgroup analyses of women with HPV 16/18 lesions prior to LEEP, the vaccine effectiveness increased to 70% against recurrence (2.5% vaccinated versus 8.5% unvaccinated, *p* < 0.05). For patients with non-HPV 16/18 prior to LEEP, 2.6% of vaccinated and 5.7% of unvaccinated patients had recurrent disease, but this difference was not statistically significant. Ghelardi 2018 looked at the distribution of HPV genotypes in women with recurrent CIN2+ lesions (N = 2/174 vaccinated and 11/176 unvaccinated) [31]. In the 4vHPV vaccinated group, the two recurrent lesions were positive for HPV 33 and 82, respectively, for a vaccine effectiveness of 100% in this sub-population. In the unvaccinated group, 81.8% of recurrent disease was HPV16/18(+). Del Pino 2020 found that for patients with HPV 16/18 prior to conization, the incidence of persistent or recurrent HSIL was 2.1% for vaccinated and 8.9% for unvaccinated patients, while for patients with non-HPV16/18 prior to conization, the incidence of persistent or recurrent HSIL was 5.1% for vaccinated and 12.5% for unvaccinated patients [28]. Statistical significance testing was not performed. The results from Sand 2020 were not significant.

Two studies assessed non-cervical outcomes (Table 2) [30,42]. In the study of women with pre-existing vulvar precancer, no significant difference was found between vaccinated and unvaccinated women for HSIL recurrence [30]. When the analysis was restricted to patients with incident or reactivated cases (defined as a negative followed by a positive HPV test), recurrence was observed in 5% of the vaccinated and 22% of the unvaccinated patients (*p* = 0.01, vaccine effectiveness 78.5%). As with prior cervical disease, the adjustment for residual disease influenced the vaccine effectiveness. Among women with a pre-existing diagnosis of genital warts, the vaccine effectiveness (95% CI) among women who had received at least one dose of 4vHPV was shown to be 89.0% (38.6–98.0) for genital warts recorded ≥1 year after immunization (“certain”) and 74.0% (16.8–91.9) when also including cases of genital warts recorded in the same year as immunization (“probable”) [42].

### 3.5. MSM

The effectiveness of HPV vaccination in MSM was assessed in three studies: two in HIV(–) MSM [46,47] and one in HIV(+) MSM (Table 3) [48]. Among HIV(–) MSM with prior biopsied and treated high-grade anal intraepithelial neoplasia (HGAIN), Swedish 2012 reported a vaccine effectiveness for subsequent HGAIN of 58% at 1 year (*p* = 0.01) and 50% at two years (*p* = 0.05) [46]. Among patients with a positive oncogenic HPV test, vaccine effectiveness was 60% for HGAIN over one year (*p* = 0.01) and 53% at 2 years (*p* = 0.05). Swedish 2014 described 4vHPV vaccine effectiveness in HIV(–) MSM with no history of anal warts or history of treated warts, now recurrence-free [47]. An estimated vaccine effectiveness of 55% was reported for recurrent anal warts. There was no association between vaccination and new anal warts among patients with a history of warts within the previous 5 years.

Thompson 2018 also looked at anal precancer endpoints, but in HIV(+) MSM. The overall study population (N = 314) included seven (2%) transwomen although it is unclear if any of these transwomen were included in the analyzed sample (N = 267/314, 85%) [48]. As vaccine and cytology data were only available for 249 of the 314 participants and no subgroup analyses were performed, it is unclear whether any transwomen were included in the outcome data reported, which did not demonstrate effectiveness. Of note, this study did not report exact follow-up values and did not include the timing of vaccination or the number of vaccine doses in the outcome analyses.

Five retrospective studies assessed the 4vHPV impact in MSM, all with anogenital warts as the sole reported disease outcome (Table 3) [23,25,27,29,36]. All studies included women and four of the five studies also included heterosexual men; therefore, MSM were only a sub-population of those studied. Only Lukac 2020 described sample size and patient age specifically for MSM subjects [36]. Reported declines in anogenital warts among the MSM population in these studies were highly varied and depended on the age as well as the sexual orientation of the men and the time period that was studied. Three studies were conducted in Australia, where MSM were not included in the HPV National Immunization Program until 2018 [23,27,29]. Similarly, boys and MSM in the United Kingdom were not eligible for HPV vaccination until 2018, after the time period studied by Checchi 2019 [25]. Lukac 2020 compared birth cohorts born 1988–1999 and 1985–1987; MSM born in 1989 or later were offered free catch-up vaccinations in British Columbia, Canada in 2015 [36,53].

### 3.6. Immunocompromised or Immunosuppressed Individuals

The effectiveness of HPV vaccination on abnormal cytology and cervical precancer in immunosuppressed/immunocompromised individuals was described in two studies from the United States, one in perinatal HIV-infected youth [40] and one in immunosuppressed women [45]. Neither study demonstrated vaccine effectiveness [40,45]. In addition, neither study reported follow-up times for study outcomes and Silverberg 2020 also did not specify vaccine doses or vaccination timing with respect to outcome assessment, nor did they stratify based on the underlying mechanism of immunosuppression, which in the study population included prior HIV infection, solid organ transplant, or a recent prescription of immunosuppressive medication [45].

### 3.7. Evidence Strength across the High-Risk Populations

This SLR found that HPV vaccination effectiveness varied across high-risk populations. Notably, study design issues and limited data contributed to this variance and the evidence strength was graded as high only for RRP patients and women with prior cervical disease (Table 4). In HIV(+) MSM and in other immunosuppressed/immunocompromised patients, issues were identified regarding study design and other aspects of reporting, including a lack of HPV genotype information in HPV-related outcomes, cytological but not histological findings reported, a lack of clarity on the timings of vaccination versus outcome observance, a lack of reporting on follow-up time, and, for impact studies in MSM, assessments prior to inclusion in a national immunization program.

## 4. Discussion

Vaccine efficacy data from clinical trials have demonstrated that 4vHPV and 9vHPV vaccines protect against HPV-related disease and that the protection is long-lasting [54,55,56]. However, clinical trials are highly controlled and often do not translate into the real-world, thus the need for real-world evidence to support the clinical trial data. Real-world evidence assessing the effectiveness and impact of these vaccines on the primary vaccinated and catch-up cohorts has demonstrated sustained and strong protective effects [5,6]. Missing from these previously published studies were several high-risk populations, some of which are either covered under national immunization programs or under consideration for such funding. The present systematic literature review sought to gather and synthesize the data on the real-world impact and effectiveness of 4vHPV/9vHPV in six high-risk populations. We found evidence that the effectiveness of 4vHPV/9vHPV vaccines is strongest for those receiving the vaccine for the prevention of subsequent disease, specifically women with prior cervical disease and RRP patients. This is in line with the findings of previous reviews that assessed HPV vaccine effectiveness against recurrence of cervical disease and/or RRP [9,10,12,14,15]. Most studies assessed 4vHPV, which was expected as 4vHPV has been available for 8 years more than 9vHPV, and real-world impact and effectiveness studies require time for outcomes to manifest. In addition to a dearth of 9vHPV real-world impact and effectiveness studies, significant data gaps remain regarding both populations at increased risk for HPV-related disease and specific disease outcomes. No studies assessed vaccine effectiveness or impact related to head and neck cancers, penile cancers, or vaginal cancer. The strength of the evidence on vaccine effectiveness and impact among MSM, HIV(+) populations and other immunocompromised or immunosuppressed individuals is weak. No real-world evidence was found for vaccine effectiveness and impact among transgender and non-binary individuals or female sex workers, highlighting the need for information on these vulnerable populations.

Although they are more complicated to design and execute, vaccine effectiveness studies, which directly compare vaccinated to unvaccinated groups, were five times more common than vaccine impact studies, which assess outcomes at the population level. Among vaccine effectiveness studies, RRP was the most highly studied outcome. RRP is a rare disease, with an estimated incidence of 4/100,000 in children and 2/100,000 in adults [18]. The recurrent nature of the disease was central to the unique design of the RRP effectiveness studies, in which individuals served as their own comparators pre- versus post-vaccination. There was evidence for an increase in the intersurgical interval and a decrease in surgeries per month following vaccination, although there was a lack of consistency in reporting statistical significance testing across the studies and low power due to small sample sizes. The number of studies devoted to this rare disease underscore its lifelong impact on patients and families and the dedication of the clinicians caring for them to alleviate their suffering.

In contrast to RRP, cervical cancer is not a rare disease. Cervical cancer is the fourth most common cancer among women worldwide with an estimated age-standardized incidence of 13.1 per 100,000 women [57]. Geographic disparities are highly pronounced with incidence rates as high as 40–80 per 100,000 women in some African nations, particularly those in sub-Saharan Africa where HIV is also prevalent [57]. In fact, women living with HIV have a six-fold higher risk of invasive cervical cancer and 5% of all cases of invasive cervical cancer in 2018 were attributed to HIV [58]. Despite this well-known increase in risk, we found no vaccine effectiveness or impact studies in women living with HIV. One study only assessed 4vHPV effectiveness in perinatally exposed and infected youth from the United States and did not demonstrate effectiveness against disease outcomes in this small population [40]. There is an urgent need to understand vaccine effectiveness and impact in women living with HIV and a high need for research in low-income countries, such as African nations, where HIV and HPV are both highly prevalent.

The most frequently studied female population was women who had undergone excisional therapy for cervical dysplasia. High-grade evidence supported vaccine effectiveness against subsequent cervical disease in this population. The vaccination of women undergoing excisional therapy, which was found to be effective in five of the six studies included in the risk population, is on label as it aims to prevent new infection among these women. Of note, the evidence here also highlights the complexity of doing real-world impact and effectiveness research. While registry databases are often considered the highest quality data sources for real-world impact and effectiveness studies, there are limitations to using registry databases [59]. For example, Sand 2020 performed a registry study using the Danish Pathology Databank with vaccination status extracted from the Danish National Prescription Registry [44]. Sand 2020 could not adjust for positive margins because it extracted data from a registry, and therefore the authors could not account for residual disease, a key confounder in studies attempting to assess effectiveness at preventing new infection.

The challenges of studying the real-world impact and effectiveness of HPV vaccines might contribute to the striking data gaps uncovered herein. In addition to the lack of studies noted above for people living with HIV, no studies of transgender and non-binary individuals were found. Transgender men with a cervix, who make up a sizable proportion of transgender men, face tremendous barriers to HPV screening and care and are absent from HPV vaccine research [60]. Likewise, female sex workers are a high-risk population about whom little is known. As with populations, there were data gaps in outcomes assessed. Cervical cancer was the most well studied HPV-related cancer, which is notable in that the non-cervical cancer burden surpassed the cervical cancer burden in the United States in 2013 [61]. The one non-cervical cancer study in women assessed vulvar precancer, a gender-specific cancer. The most common non-cervical cancer, anal cancer, which is more common in women than men, was only studied in MSM. The lack of organized screening programs for non-cervical cancers may contribute to this evidence gap.

There are several limitations with the present SLR. The review was limited by the heterogeneity of definitions, study designs, and outcome reporting. The studies we reviewed of vaccine effectiveness among HIV(+) MSM and immunocompromised individuals were weakened by study design and reporting issues noted above relating to the lack of HPV genotyping, the lack of reporting of vaccination timing or follow-up time, and the insufficient characterization of outcomes. Previous SLRs have reported similar issues with the quantity and/or quality of evidence for HPV vaccine effectiveness in these populations [11,16]. Further, there was inconsistent reporting of potential confounders and few studies provided statistical analyses of comparisons between groups. The vaccine effectiveness and impact estimates provided are therefore based on a limited number of comparisons, do not include error values in many cases, and are not derived from a quantitative synthesis across studies (meta-analysis). Furthermore, in some vaccine effectiveness studies of women with pre-existing cervical or vulvar disease and in both studies in HIV(-) MSM, individuals were charged for HPV vaccines, which may have introduced bias [28,31,46,47]. All vaccine impact studies included some time periods when males were not included in the national immunization programs, requiring patients to pay for vaccines in those years which, likewise, may have introduced bias [23,25,27,29,36]. Studies that included the use of 2vHPV in the majority of vaccinated subjects were excluded. While 2vHPV is effective against cervical disease, it does not provide direct protection against the HPV6- and HPV11-related diseases assessed herein (anogenital warts, RRP). Other published reviews that address cervical precancers/cancer as the sole outcome include data on 2vHPV’s efficacy against cervical disease [62].

Balancing these limitations are the strengths of this SLR, including its focus on real-world evidence and clinical outcomes rather than HPV test positivity alone, as test positivity does not necessarily correlate with disease outcomes of interest to patients and public health decision makers. We also included a more diverse set of disease outcomes than most previous SLRs. A third strength is the compilation of data on different high-risk populations, which have heterogeneous characteristics, needs, and prevalence of various HPV-related diseases, which are currently included in or under consideration for inclusion in national or regional immunization programs.

## 5. Conclusions

In conclusion, the findings of this SLR support the effectiveness of 4vHPV/9vHPV vaccination, primarily 4vHPV, in preventing subsequent cervical precancer in women with pre-existing cervical disease, and in improving clinical outcomes in patients with RRP. In HIV(–)MSM, there was also evidence of moderate effectiveness of 4vHPV in preventing subsequent HGAIN and new or subsequent anal warts in those with and without pre-existing disease. Conspicuous data gaps in populations and in HPV-related outcomes were uncovered. The evidence for the effectiveness and impact of HPV vaccination in several vulnerable high-risk populations was either absent or unclear, although a number of these populations (MSM, transgender) are either covered under some immunization programs or under consideration for such funding. Further investigation is warranted to understand the public health implications of HPV vaccination for these groups and to bolster public health decision making for populations at an elevated risk of preventable HPV-related disease.

## Figures and Tables

**Table 1:** PICOTS (population, interventions, comparisons, outcomes, timing, and setting) criteria for the systematic literature review.

	Inclusion Criteria	Exclusion Criteria
**Populations**	People of any age, from any country, considered at high risk for HPV-related disease, including:∙ People with recurrent respiratory papillomatosis∙ Women with pre-existing HPV-related anogenital disease∙ Men who have sex with men∙ Immunocompromised or immunosuppressed individuals, including those positive for HIV or who have received immunosuppressive treatments (such as those receiving transplants)∙ Transgender and non-binary individuals∙ Sex workers	People not in one of the identified high-risk populations
**Interventions**	HPV vaccination	Use of 2vHPV in >50% of vaccinated subjects
**Comparisons**	No vaccine (effectiveness) Before vaccination programs implemented (impact)	
**Outcomes**	Incidence or prevalence of genital warts or HPV-related precancer or cancer, including:∙ Genital warts∙ Precancers or cancer of the cervix (including low- and high-grade cervical lesions)∙ Precancers or cancer of other anogenital regions (including vulva, vagina, penis, or anus)∙ Precancers or cancer of the oral cavity, larynx, or oropharyngeal regions∙ Recurrent respiratory papillomatosis	Patient-reported outcomes (e.g., quality of life, satisfaction) Economic outcomes (e.g., cost-effectiveness, budget impact) Population level incidence/prevalence of Juvenile onset RRP (JoRRP vaccine impact studies)
**Time**	Conference abstracts: 1 January 2018 to present Articles: any time	Conference abstracts published before 2018
**Study design**	Observational studies using real-world data (e.g., longitudinal survey, medical records, registry) Systematic reviews (for identification of primary studies only)	Randomized trials, including long-term follow-up studies of clinical trial cohorts Decision Analytic and cost-effectiveness modeling/simulation studies Non-systematic reviews Editorials, comments, notes Case studies Clinical practice guidelines
**Other**	English language	Non-English language

HIV, human immunodeficiency virus; HPV, human papillomavirus; 2vHPV, bivalent HPV vaccine; 4vHPV, quadrivalent HPV vaccine; 9vHPV, nonavalent HPV vaccine.

**Table 2:** Vaccine effectiveness studies in women with prior anogenital disease.

Author Year	Country	Patient Description	Demonstrated Effect ^a^
**Cervical Disease**			
Bogani 2020 [24]	Italy	Women undergoing conization for cervical HSIL, 60 months minimum follow-up	69% in those HPV-free at 6 months to adjust for residual disease. NS in total population.
Del Pino 2020 [28]	Spain	Women undergoing conization between January 2013 and July 2018. Mean 22.4 months follow-up	80% in total population. For those disease-free at 6 months (negative HPV test, negative Pap test, and, if available, a negative biopsy), 100% for HSIL.
Ghelardi 2018 [31]	Italy	Women undergoing conization for cervical HSIL/CIN2-3, 36 month median follow-up	81.2% in analyses adjusting for residual disease. NS without adjustment for residual disease.
Kang 2013 [35]	South Korea	Women aged 20–45 years undergoing LEEP for histologically confirmed CIN2-3, 42 month median follow-up, 2 year minimum	65% in those disease-free at 3 months, ^b^ 70% among those with vaccine type lesions before LEEP who were disease-free at 3 months.
Petrillo 2020 [43]	Italy	Women undergoing LEEP for CIN1 or greater	76% for CIN2+.
Sand 2020 [44]	Denmark	Women undergoing conization for CIN3 or greater	NS
**Vulvar Precancer**			
Ghelardi 2021 [30]	Italy	Women undergoing surgical treatment for vulvar HSIL	NS overall: 78.5% for disease from incident/reactivated infection.
**Genital Warts**			
Petráš 2015 [42]	Czech Republic	Women aged 16–40 years with prior history of genital warts ^c^	For 4vHPV: 89.0% for certain cases, 74.0% for possible and certain cases.

HPV, human papillomavirus; NS, not significant. ^a^ Reported for multivariable analyses when available. ^b^ Women with positive histology of colposcopy-directed biopsy or endocervical curettage 3 months after LEEP are considered to have residual CIN 2-3. ^c^ Patients with no prior genital warts were not within the scope of this SLR. Reporting is restricted to women with a prior history of genital warts (N = 175).

**Table 3:** Summary of vaccine effectiveness and impact studies in MSM.

Author Year	Country	Patient Description	Demonstrated Effect ^a^
Effectiveness Studies			
Swedish 2012 [46]	United States	HIV(−) MSM with prior biopsied and treated HGAIN	58% for recurrent HGAIN at 1 year and 50% at 2 years. Among patients HPV DNA(+) for high-risk genotypes, 60% at 1 year and 53% at 2 years. NS at 3 years for all comparisons.
Swedish 2014 [47]	United States	HIV(−) MSM ≥26 y with no anal condyloma history or previously treated, recurrence-free condyloma	55% in total population. NS for recurrent AGW among patients with history of AGW within the past 5 years.
Thompson 2018 [48]	United States	HIV(+) MSM (98%) and transwomen (2%) ^b^	NS against anal precancers among 85% of the population with anal cytology data.
**Impact Studies of 4vHPV against Anogenital Warts**			
Ali 2017 [23]	Australia	Indigenous and non-indigenous MSM attending 39 sexual health clinics ^c^	36% decline in rate for non-Indigenous. NS for Indigenous between 2004 and 2007 and between 2008 and 2014.
Chow 2015 [27]	Australia	MSM aged 16–40 years attending 1 sexual health clinic ^d^	33% decline overall between 2004 and 2005 and between 2013 and 2014. Variation by age, sexual orientation, and wart location was noted.
Donovan 2011 [29]	Australia	MSM attending 8 sexual health clinics ^d^	28% decline between January and June 2004 and between January and June 2007.
Checchi 2019 [25]	United Kingdom	MSM aged 15–24 years attending sexual health clinics participating in national surveillance system ^d^	79% decline for 15 year olds between 2014 and 2017. NS for other age groups (15–17, 18–20, and 21–24).
Lukac 2020 [36]	Canada	MSM aged 14–46 years attending 16 sexual health clinics ^d^	41% decline between 1991 and 1993 and between 1994 and 1996. NS for earlier birth cohort comparisons.

AGW, anogenital warts; HIV, human immunodeficiency virus; HPV, human papillomavirus; MSM, men who have sex with men; NS, not significant. ^a^ Reported for multivariable analyses when available. ^b^ There were N = 7 transwomen included in overall cohort of N = 314 subjects, but only N = 267 had anal screening, and since no subgroup analyses were performed, it is unclear if any data in this report were from transwomen. ^c^ The study also included Indigenous women, who are not within the scope of this SLR. ^d^ The study also included women and heterosexual men, who are not within the scope of this SLR.

**Table 4:** Strength of evidence ratings and rationale ^a^.

Design and Population	Strength of Evidence ^b^	Study Limitations ^c^	Directness ^d^	Consistency ^e^	Precision ^f^	Publication Bias ^g^
VACCINE EFFECTIVENESS STUDIES						
RRP patients ^h^	High	Medium	Direct	Consistent	Imprecise	Low
Women with pre-existing anogenital disease						
Cervical disease	High	Medium	Direct	Consistent	Precise	Low
Vulvar precancer	Medium	Low	Direct	NA ^i^	NA ^i^	Low
Genital warts ^j^	Medium	Medium	Direct	NA ^i^	NA ^i^	Low
MSM						
HIV(–) ^k^	Medium	Medium	Indirect	NA ^i^	NA ^i^	Low
HIV(+) ^l^	Low	High	Direct	NA ^i^	NA ^i^	Low
Immunosuppressed/ Immunocompromised individuals ^m^	Low	High	Indirect	NA ^i^	NA ^i^	Low
VACCINE IMPACT STUDIES						
MSM ^n^	Low	High	Direct	Consistent	Precise	Low

HIV, human immunodeficiency virus; MSM, men who have sex with men; NA, not applicable; RRP, recurrent respiratory papillomatosis. ^a^ Adapted from Grading of Recommendations, Assessment, Development and Evaluation (GRADE) Working Group [22]. ^b^ Based on the answers in the study limitations, directness, consistency, precision, and reporting bias domains. ^c^ Refers to whether studies have a high likelihood of protection against bias. Limitations for observational studies include (1) failure to develop and apply eligibility criteria, (2) flawed outcome measurement, (3) failure to control confounding factors, and (4) incomplete/inadequate follow-up. ^d^ Refers to (1) differences across populations, (2) differences in interventions, (3) differences in outcome measures/surrogate outcomes, and (4) indirect comparisons (not applicable in this instance). ^e^ Refers to whether the included studies find the same direction or magnitude of effect. ^f^ Refers to the degree of certainty based on the studies’ size and event number. ^g^ Refers to selective publication of findings based on direction or magnitude of effect favorability. There was no evidence to suggest publication bias for any results. ^h^ Limitations rated as high due to differences in how partial response was determined; imprecise rating due to relatively small sample sizes in all studies. ^i^ Not applicable due to the results for the respective population and outcome being drawn from a single study. ^j^ Limitations rated as medium due to the survey-based nature of the single study. ^k^ Limitations rated as medium due to the cost for patients to receive the vaccine; indirect rating due to the different outcomes assessed among these two studies; inconsistent/imprecise ratings due to the different outcomes assessed in the two studies. ^l^ Limitations rated as high due to confounding factors such as under-vaccinated participants, lack of reporting of follow-up time or timing of vaccination versus outcomes; imprecise ratings due to single study. ^m^ Limitations rated as high due to all studies having limitations (lack of follow-up time, cytological instead of histological outcomes, and/or other confounding factors); indirect rating due to the different populations included; inconsistent rating due to different outcomes measured by the two studies; and imprecise rating due to small sample sizes and low proportions of events. ^n^ Limitations rated as high due to the ineligibility of MSM for National Immunization Program in four of the five studies.

## Supplementary Materials

The following supporting information can be downloaded at: https://www.mdpi.com/article/10.3390/vaccines10091543/s1, Table S1: Search strategy in MEDLINE/Embase (via ProQuest).
