# Correction: Goodman et al. Human Papillomavirus Vaccine Impact and Effectiveness in Six High-Risk Populations: A Systematic Literature Review. *Vaccines* 2022, *10*, 1543

**DOI:** 10.3390/vaccines11071227

**Published:** 2023-07-11

**Authors:** Elizabeth Goodman, Miriam Reuschenbach, Allysen Kaminski, Sarah Ronnebaum

**Affiliations:** 1 Center for Observational and Real-World Evidence, Merck & Co., Inc., Rahway, NJ 07065, USA; 2 Global Medical and Scientific Affairs, MSD Sharp and Dohme GmbH, 81673 Munich, Germany; miriam.reuschenbach@msd.de; 3 OPEN Health, Bethesda, MD 20814, USA; akaminski@gwmail.gwu.edu (A.K.); sarah.ronnebaum@evidera.com (S.R.)

The authors wish to make the following corrections to this paper [1]. In the original article, there was a mistake in Figure 2 as published. A few of the references were missed from the legend for Figure 2B,C. The corrected Figure 2 appears below.

The authors state that the scientific conclusions are unaffected. This correction was approved by the Academic Editor. The original publication has also been updated.

## Figures and Tables

**Figure 2:**
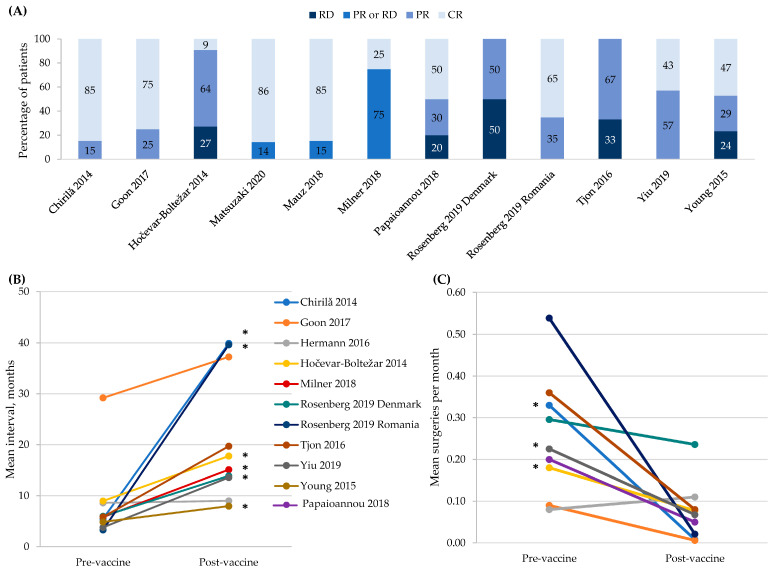
4vHPV vaccine effectiveness in patients with recurrent respiratory papillomatosis. Panel (**A**) shows the tumor response within an individual patient defined as: CR, complete response; PR, partial response; and RD, recurrent disease. Three studies defined PR as a >50% increase in the interval between surgical procedures [32,49,50]; one study as a >50% increase in the interval between surgical procedures or persistent papillomas that were not growing and did not require surgical interventions for >12 months [34]; one study as any increase in the time to recurrence [41]; and one study as >12 months with no appreciable growth of papillomas [51]. Three studies defined the ‘PR or RD’ category as any recurrence [37–39]. No definitions were provided in the remaining two studies [17,26]. These are descriptive, within person responses, so vaccine effectiveness is not calculated. Panel (**B**) shows the increase in the mean intersurgical interval. The symbol * indicates statistical significance at *p* < 0.05. Panel (**C**) shows the decrease in the mean number of surgeries per month. The symbol * indicates statistical significance at *p* < 0.05. For all panels, data from the Rosenberg 2019 Danish cohort were collected by the study authors while data from the Romanian cohort were new, never reported data from a prior published study [52].
