# Supplementary material for: Human Papillomavirus Vaccine Impact and Effectiveness in Six High-Risk Populations: A Systematic Literature Review
Source: Vaccines (Basel). 2022 Sep 16;10(9):1543. doi: 10.3390/vaccines10091543 (PMC9503207; doi:10.3390/vaccines10091543)
Supplement: Supplementary file 1 [file vaccines-10-01543-s001.zip › vaccines-1858270-supplementary.pdf]

SUPPLMENTARY TABLE

Supplementary Table S1. Search strategy in MEDLINE/Embase (via ProQuest)

Search	Terms	Studies identified
S1	TI,AB(((“papillomavirus” OR “papillomaviruses” OR HPV) NEAR/1 (vaccine OR vaccination)) OR Gardasil OR Silgard OR HPV4 OR 4VHPV OR HPV9 OR 9VHPV) OR MESH.EXACT(“Human Papillomavirus Recombinant Vaccine Quadrivalent, Types 6, 11, 16, 18”) OR EMB.EXACT(“Wart virus vaccine”)	28,683
S2	TI,AB(((risk OR risky OR susceptible OR susceptibility OR likelihood OR likely) NEAR/1 (high OR higher OR highly OR elevated OR increased OR greater OR more)) OR "high-risk" OR "disease cofactor" OR HIV OR "HIV-positive" OR "human immunodeficiency" OR "HPV-positive" OR conization OR "cone biopsy" OR "electrosurgical excision" OR adjuvant OR prophylactic OR “catch-up” OR MSM OR “men who have sex with men” OR "males who have sex with males" OR “gay men” OR transgender OR non-monogamous OR immunocompromised OR immunosuppressed OR "sex worker" OR "prostitution" OR prostitutes OR dropout OR drop-out) OR MESH.EXACT(“Unsafe Sex” OR “Sex Work” OR “Student Dropouts”) OR MESH.EXACT.EXPLODE(HIV OR “Sexual and Gender Minorities” OR “Immunocompromised Host”) OR EMB.EXACT(“sex worker” OR prostitution OR “school dropout”) OR EMB.EXACT.EXPLODE(“unsafe sex” OR “human immunodeficiency virus” OR “sexual and gender minority”)	4,274,018
S3	TI,AB(effectiveness OR impact OR incidence OR prevalence OR CIN1 OR "CIN 1" OR CIN2 OR "CIN 2" OR CIN3 OR "CIN 3" OR adenocarcinoma OR LSIL OR HSIL OR “recurrent respiratory papillomatosis” OR RRP OR “laryngeal papillomatosis” OR precancerous OR precancer) OR TI,AB((“head and neck” OR oral OR cervical OR cervix OR genital OR anogenital OR penile OR anal OR anus OR vulva OR vaginal OR vagina OR oropharyngeal) NEAR/3 (abnormalit[*4] OR lesion OR lesions OR neoplasia OR neoplasias OR neoplasm OR neoplasms OR adenocarcinoma OR adenocarcinomas OR dysplasia OR dysplasias OR warts OR cancer OR cancers OR carcinoma OR carcinomas)) OR MESH.EXACT(“Papillomavirus Infections” OR "Cervical Intraepithelial Neoplasia" OR “Uterine Cervical Neoplasms” OR “Vaginal Neoplasms” OR “Vulvar Neoplasms” OR “Penile Neoplasms”) OR MESH.EXACT.EXPLODE(“Head and Neck Neoplasms” OR “Condylomata Acuminata”) OR EMB.EXACT(“human papillomavirus infection” OR "larynx papillomatosis") OR EMB.EXACT.EXPLODE(“uterine cervix cancer” OR “vagina cancer” OR “vulva cancer” OR “penis cancer” OR “head and neck cancer” OR “condylomata acuminata”)	7,856,561
S4	TI,AB((population OR sentinel OR cohort OR "follow up" OR observational) NEAR/1 (surveillance OR survey OR study OR studies OR analys*)) OR TI,AB(“case control” OR "program evaluation" OR longitudinal OR retrospective OR “cross sectional” OR “cross-sectional” OR “epidemiologic*” OR registry OR registries OR database OR databases OR chart	11,625,982

Search	Terms	Studies identified
	OR charts OR claim OR claims OR record OR records OR “real world” OR real-world OR "real life" OR "real-life" OR “medical record” OR “health record” OR extension OR “long term” OR “long-term” OR "pre-vaccination" OR “pre-vaccine” OR "post-vaccination" OR “post-vaccine” OR "before and after") OR MESH.EXACT(“Epidemiologic Studies” OR “Cross-Sectional Studies” OR “Insurance Claim Review” OR “Electronic Health Records” OR “Observational Study” OR "Program Evaluation") OR MESH.EXACT.EXPLODE(“Case-Control Studies” OR “Cohort Studies” OR Registries OR "Population Surveillance") OR EMB.EXACT(“case control study” OR “family study” OR “longitudinal study” OR “retrospective study” OR (“prospective study” NOT “randomized controlled trials”) OR “cohort analysis” OR “observational study” OR “cross-sectional study” OR “disease registry” OR “administrative claims (health care)” OR “medical record review” OR “medical record” OR "health survey" OR "sentinel surveillance") OR EMB.EXACT.EXPLODE("program evaluation")	
S5 [a]	S2 AND S3 AND S4 AND S5	2,635
S6	EMB.EXACT(“case study” OR “case report” OR “abstract report” OR “letter” OR “note”) OR DTYPE(“Letter” OR “Historical Article” OR “Editorial” OR “Note” OR “Comment” OR "News" OR "Newspaper Article" OR “Guidelines” OR “Practice Guideline” OR “Consensus Development Conference” OR “Consensus Development Conference, NIH”) OR TI,AB(“case study” or “case studies” OR "case report" OR "case reports" OR “case series”)	9,039,120
S7 [a]	S5 NOT S6	2,537
S8 [a]	(S7 AND PD(>2017) AND DTYPE("Conference abstract")) OR (S7 NOT DTYPE("Conference abstract"))	2,200
